# Does Population Aging Impact China’s Economic Growth?

**DOI:** 10.3390/ijerph191912171

**Published:** 2022-09-26

**Authors:** Qiuxing Chen, Qiaozhu Chi, Yang Chen, Oleksii Lyulyov, Tetyana Pimonenko

**Affiliations:** 1 School of Economics, Fujian Normal University, Fuzhou 350117, China; 2 School of Finance and Accounting, Fujian Business University, Fuzhou 350500, China; 3 Department of Marketing, Sumy State University, 40007 Sumy, Ukraine

**Keywords:** population aging, endowment insurance expenditure, medical and health care expenditure, economic growth

## Abstract

The rapid aging of the population presents great challenges in terms of China’s social security expenditure and economic growth. This paper uses the entropy method to comprehensively measure the provincial population aging index in 2008–2019 and constructs an intermediary effect model with it as the core explanatory variable. The results show that the population aging has a significant positive impact on economic growth and on the promotion of the economic growth of more developed areas; it also has a positive impact on the endowment insurance expenditure and medical and health expenditure and on the promotion of economically backward areas. Endowment spending and health spending fully mediate the relationship between population aging and economic growth.

## 1. Introduction

Since the reform and opening, with the rapid economic development of China’s population and the great progress made in science and technology, the life expectancy of residents has continued to grow. In 1990, the average life expectancy of China’s population was 68.6 years, and it reached 76.4 years in 2018, an increase of 7 years [1]. In addition, the implementation of the family planning policy for many years has greatly reduced the birth rate of infants, and the reduced proportion of children and the extension of the elderly’s life expectancy has caused the severe aging situation of China’s population. Statistics show that, in 2000, China’s population aged 65 and above accounted for 7% of the total population [1]. According to international standards, China has become an aging society. By 2021, the proportion will increase to 14.20%, and the population aging coefficient has about doubled compared with 2000, confirming this reality [1].

The impact of population aging on China’s social and economic growth is mainly reflected in two aspects. On the one hand, the change in population age structure leads to the shortage of effective labor supply, which reduces the production efficiency of enterprises and is not conducive to long-term and stable economic development [2,3,4,5,6,7,8]. On the other hand, population aging requires more social security expenditure, and the continuous expanding pension fund gap places great pressure on local governments [9,10,11,12,13,14]. However, it should not be ignored that population aging also forces enterprises to improve technological innovation and increase social security spending when reducing labor supply, so it could drive economic growth [15,16,17]. Therefore, it cannot be easily concluded whether the inhibitory impact of population aging on economic growth is positive or negative.

Based on the above background, this paper puts forward the following three questions: First, does the aging population of China’s economic growth have a promoting or hindering effect? Second, what is the role of endowment insurance expenditure and medical insurance expenditure in the impact mechanism of population aging on economic growth? Third, what are the differences in the impact of population aging on economic growth in different regions? Grasping the mechanism of the influence of population aging and social security expenditure on economic growth is of practical significance to promote the benign interaction between social security expenditure and economic growth under the background of deep aging.

This paper has the following structure: a literature review, i.e., an analysis of the theoretical framework of the impact of population aging on China’s economic growth to highlight the hypothesis of the investigation; methods, i.e., explanations of the applied methods to investigate the paper’s hypothesis; results, i.e., explanations of the empirical results of the analysis; discussion and conclusion, i.e., descriptions of the core findings and recommendations for policy improvements in the sphere of the population aging. 

## 2. Literature Review

### 2.1. Population Aging and Economic Growth

The impact of population aging on economic growth can be examined from both the supply side and the demand side. On the supply side, on the one hand, aging has a labor force effect. Zhang et al. [18] pointed out that China’s new stage of development has experienced a transition from a demographic dividend to population debt, and the scarcity of labor force and the rising dependency ratio of the elderly have brought certain challenges to the economy. Zhu et al. [19] pointed out that, with the increased aging of the population, the increasing number of elderly people and the increasing pressure on the young population will reduce the health level of residents, reduce the quality of the labor force, and have a negative impact on the labor market. In addition, these issues could be aggravated by the consequences of COVID-19 [20,21]. However, some scholars put forward different views. Xie X. et al. [22], using panel data from 31 provinces in China from 1998–2017 for empirical analysis, found that an aging population can promote technological innovation, and the beneficial effect of technological innovation is greater than the adverse effect of labor reduction, so the aging population has no obvious negative effect on China’s economic growth in China.

On the other hand, the aging population has a savings effect. According to the life cycle theory, young individuals will tend to save part of the income for retirement. After individuals are over a certain age, their savings rate will decline. As the aging population increases, the overall social savings rate will decline. An aging population entails a lower savings rate that will threaten economic development in the long term [23]. Some scholars have put forward different views. Wang et al. [24] constructed a three-generation overlapping model considering the life extension effect and social burden effect of population aging. The study found that the negative impact of the social burden effect caused by the rise in the elderly population on the savings rate was not significant, while the positive impact of the life extension effect was. Savings can accumulate idle social funds to invest in large projects, thus promoting economic development.

From the demand side, population aging promotes the increase of medical expenses and promotes the demand for elderly care services [25,26,27,28,29,30,31,32]. Although Cai et al. [25] confirmed that the labor participation rate of the elderly is low, the labor income effect decreases rapidly, and their consumption level will shrink with the increase of age [25], and the increasing degree of the aging of China’s population. The inhibition of household consumption rate is increasingly significant [26]. However, these studies ignore the fact that consumption age is a key factor of consumption structure [27,28,29,30], because the elderly’s consumption habits are particular. The increase of the elderly population will lead to a new consumer demand structure, although the elderly demand for fashion clothing, jewelry, and other luxuries is relatively weak. However, for nutrition care and longevity pension products, demand is relatively large, leading to new opportunities for the development of the silver economy. Ma et al. [31] found that population aging can promote the transfer of the labor force to the advanced industrial sector, which is conducive to the development of the advanced industry. Fang W. [32] found through empirical analysis that China’s population aging promotes the transformation and upgrading of industrial structure. The rationalization and upgrading of the industrial structure can effectively promote economic development. Thus, considering it:

**Hypothesis** **1.** *China’s population aging has a positive role in promoting economic growth.*

### 2.2. Population Aging, Endowment Insurance Expenditure, and Economic Growth

Practice shows that the development history of China’s social security reflects the role of China’s social security in unleashing the potential of economic growth, and that this growth in turn promotes the development of the social security system [33]. The improvement of the social security system has a positive role in eliminating poverty and promoting residents’ consumption, and can effectively promote economic growth [34]. Various expenditure items of social security expenditure have a different effect on economic growth. In the serious stage of population aging in China, social security expenditure and medical and health expenditure have a crowding-out effect on other expenditures [35]. The following will be mainly analyzed from two aspects: pension expenditure and health expenditure.

With the growth of the average life expectancy of the Chinese population and the aggravation of population aging, individuals increasingly receive a pension, and the endowment insurance expenditure increases year by year. Liu X. [36], through building an economic model, shows that population life expectancy growth will increase pension fund spending. As an important source of economic development, pension fund assets are important for long-term investment in less developed countries [37]. Increased pension spending inevitably reduces the amount of pension retained, but pension spending can boost economic growth by stimulating consumption. Endowment insurance fund pay is actually indirect savings for people in the future, which decreases residents’ disposable income in the current period, but national endowment insurance spending through an endowment insurance fund will be redistributed to residents to increase their disposable income, thus improving the stability of the group and their consumption ability, obviously conducive to economic growth. Zhang et al. [38] found through empirical research that social endowment insurance can effectively stimulate the consumption of the elderly, thus leading to improved economic benefits.

**Hypothesis** **2.** *Population aging has a positive effect on endowment insurance expenditure.*

**Hypothesis** **3.** *Population aging will affect economic growth through pension spending.*

### 2.3. Population Aging, Medical and Health Care Expenditure, and Economic Growth

Individuals’ demand for health capital increases as age increases, and the demand for medical services increases accordingly. Li et al. [39] used Beijing as an example for empirical analysis and found a significant positive relationship between aging and the growth rate of medical costs. Tian et al. [40] conducted an empirical analysis through a fixed-effect model, and the results show that population aging will significantly increase the social security, employment, and medical and health expenditure in areas with high levels of economic development. However, scholars have questioned the positive relationship between aging and medical expenses. Thus, some studies [41,42,43] suggest that the role of aging on medical costs is exaggerated, and the high medical costs paid to save lives before the end of life are greatly ignored. The question suggests that the high health costs before the end of life are the main reason for the increase in health costs. However, from another perspective, it is poor functioning and immunity that makes them more vulnerable to death. Taking Beijing as an example, in the age distribution of medical expenses, the medical expenses of the elderly aged 60 and above account for about half of the total medical expenses, indicating that the elderly population is the main consumer of social medical resources [39]. Growth in the elderly population will promote an increase in health care spending. At the same time, the differences in population development laws in different countries also mean that foreign experience is not fully applicable to China. Lopreite et al. [44] compared the demographic structure and economic trajectory of the United States and China, showing that the impact of an aging population on health spending and economic growth is more pronounced in China.

Health and health spending can affect economic growth. First, increased medical and health expenditure can improve social medical and health conditions, especially the quality of medical and health services in rural areas, so as to effectively guarantee the health level of workers and thus ensure the high-quality supply of the labor force. An empirical study by Xie et al. [45] shows that increasing government health expenditure can improve the stability and growth level of rural economic development. Second, increased government spending on health care will stimulate the expansion of demand for health services. Tao et al. [46], based on an analysis of a panel threshold regression model, found that the government health expenditure would play a positive role in promoting the residents’ medical care consumption, thus driving the growth of overall social consumption. Finally, the increase of government medical and health expenditure is conducive to promoting the development of the medical and health service industry, the expansion of employment capacity, and economic growth.

**Hypothesis** **4.** *Population aging has a positive promoting effect on health care expenditure.*

**Hypothesis** **5.** *Population aging will contribute to economic growth through health care spending.*

## 3. Materials and Methods

The existing measure of population aging generally used internationally is based on the proportion of the elderly population in the total population. This method can measure the degree of aging of a country or region, but it cannot measure the depth of population aging; the impact of population aging on the social burden caused by a region is not considered, which is often affected by the proportion of children and the proportion of labor population. Therefore, this paper constructs a measurement index system of population aging and comprehensively considers the breadth, depth, and speed of population aging in a region.

### 3.1. The Population Aging Index Measure

The entropy method is used to weight the index system to obtain the degree of population aging. This method is based on macro data and is objective and scientific compared with traditional subjective value judgments. The principle of entropy value calculation steps is as follows:

(1) Initial matrix

Assuming *n* indicators and m study samples, the initial data matrix is

(1) $$X=\left[x_{ij}\right]m\times n$$

where *X* is the initial data matrix; $x_{ij}$ is the *i*th value of the *j*th index of the system.

(2) Standardized treatment

The data in Equation (1) is standardized. If the value of an index and the evaluation result change in the same direction, it is a positive index, and the standardization formula is

(2) $$Y=\frac{\left(X_{ij}-X_{min}\right)}{\left(X_{max}-X_{min}\right)}$$

Conversely, the negative index standardization equation is

(3) $$Y=\frac{\left(X_{max}-X_{ij}\right)}{\left(X_{max}-X_{min}\right)}$$

The normalized matrix is obtained:

(4) $$Y=\left[y_{ij}\right]$$

(3) Entropy value calculation

The information entropy value of the j-item index is

(5) $$e_{j}=-k\overset{m}{\underset{i=1}{\sum }}f_{ij}\ln f_{ij,\;}\left(i=1,\;2\ldots ,m;j=1,2,\ldots ,n\right)$$

among

(6) $$f_{ij}=\frac{y_{ij}}{\sum_{i=1}^{m}y_{ij}}$$

where $e_{j}$ is the entropy value of the item *j* index; *k* is a constant, where *k* = 1/ln(*m*); $f_{ij}$ is the weight of the *i*th value of the item j index. 

(4) Weight calculation

The degree of consistency of each scheme under the *j*th attribute *d_j_* = 1 − *e_j_*. The weight formula of each attribute can be obtained as follows:

(7) $$W_{j}=d_{j}/\overset{n}{\underset{j=1}{\sum }}d_{j}$$

In this paper, through the breadth, depth, and speed of population aging, we respectively select the corresponding measurement indicators to obtain the comprehensive level of the degree of population aging. The selection of indicators is shown in Table 1.

The index data of this paper were obtained from the National Bureau of Statistics and the National Statistical Yearbook [1]. Thirty-one Chinese provinces (autonomous regions and municipalities directly under the Central Government) were selected as the research subjects and divided into eastern, central, and western regions. The provincial index data from 2008 to 2019 were collected and compiled, and the average geometric growth rate was calculated to compensate for individual missing data. 

### 3.2. Model Construction and Variable Description

The variables selected for the model construction are shown in Table 2. Thus, the explained variable is the local economic growth level. The explanatory variable is the population aging index. The intermediary variables are the pension insurance expenditure and the health care expenditure. The control variables include the urbanization rate, the population education level, and the natural population growth rate. The economic growth level is measured by the per capita GDP, and the population aging index is calculated by the entropy method.

To test the hypothesis, the following model is constructed using [47]:

VGDP_i__,__t_ = α + β_1_AoP_i__,__t_ + β_1_UBR_i__,__t_ + β_2_PEL_i__,__t_ + β_3_NPGR_i__,__t_ + ε_I_ (8)

PFE_i,t_ = α + δ_1_AoP_i__,__t_ + δ_2_UBR_i,t_ + δ_3_PEL_i__,__t_ + δ_4_NPGR_i__,__t_ + ε_i I_ (9)

MPE_i,t_ = α + ϕ_1_AoP_i__,__t_ + ϕ_2_UBR_i,t_ + ϕ_3_PEL_i__,__t_ + ϕ_4_NPGR_i__,__t_ + ε_i I_ (10)

VGDP_i,t_ = α + θ_1_AoP_i,t_ + θ_2_PFE_i,t_ + θ_3_UBR_i,t_ + θ_4_PEL_i,t_ + θ_5_NPGR_i,t_ + ε_i I_ (11)

VGDP_i,t_= α + ψ_1_AoP_i,t_ + ψ_2_MPE_i,t_ + ψ_3_UBR_i,t_ + ψ_4_PEL_i,t_ + ψ_5_NPGR_i,t_ + ε_i i_ (12)

Equation (8) tests the total effect of the relationship between population aging and economic growth. Thus, Equation (9) tests the relationship between population aging and pension fund expenditure, and Equation (11) adds intermediary variables to population aging and economic growth. First, to test Equation (8), if the coefficient is significant in Equation (7), the next step will be applied. If the coefficient d1 and q2 (Equations (9) and (11)) are significant, the mediation effect will exist. If one is insignificant, the fourth step test is required. According to the test results of the previous step, if in Equation (11) coefficient q1 is not significant, it will mean that there is a complete mediation effect. Thus, the explanatory variable to affect the explained variable should pass through the mediation variable. Otherwise, there is a partial mediation effect. The part of the influence is realized through the mediation effect. The study applied the Bootstrap sampling method. 

Multicollinearity diagnosis was performed using SPSS software, and the variance expansion factor of each variable was less than 10, indicating that there was no multicollinearity among the independent variables. Regression analysis of the samples yielded the following results for each regression.

## 4. Results

### 4.1. Population Aging Index

The aging index findings are shown in Table 3. Except for Tianjin and Tibet, where the population aging index was slightly declining (Tibet = −1.45%; Fujian province: −1.26%), an upward trend was generally found. 

Compared with 2008, the three provinces with the highest rising rate in 2019 were the Heilongjiang, Shandong, and Hebei provinces, with 63.87%, 63.52%, and 51.15%, respectively. The smallest increases were in the Tianjin (−3.57), Tibet, and Fujian provinces.

### 4.2. Difference Analysis of the Regional Population Aging Index

According to the entropy method, the population aging index in the east, central, and west regions of China can also be calculated. As shown in Table 4, the population aging index varied greatly between the three regions. The eastern index is the largest, with an average value of 0.448, followed by the central part with a mean value of 0.418 and the west with a mean value of 0.374. 

The largest coefficient of variation was in the west, followed by the eastern and central regions. The difference in the population aging index was the largest among the western provinces and the smallest in the central region. The eastern region had the largest breadth, and the provinces differed the most. In contrast, the eastern region has the smallest depth, with an average of 5.930, compared to 6.701 and 7.236 in the central and western regions. Finally, the rate of aging was almost similar in each region, with the largest differences in the western region, followed by the east. 

The secondary measures are shown in Table 5. The eastern region has the largest aging population coefficient, while the western region has the largest population ratio of children. 

One reason that the social burden in the west is heavier than that in other regions is that their children are raised for longer periods of time. The eastern region has the largest ratio of young people, i.e., 0.819, with 0.644 in the central region and 0.505 in the west. It can be seen that population aging in the eastern region is a large social burden, although the western region’s social burden is also heavy. This is largely due to the burden of children. In the long term, the western region has a sufficient labor force that can better cope with the burden of support for the elderly.

### 4.3. Test of the Intermediary Effect of the Pension Fund Expenditure

At the next step of the investigation the intermediary role of the pension fund on the relationship between population aging and economic growth was calculated. The findings are shown in Table 6. 

The findings (Table 6) show that population aging has a significant positive impact on economic growth, with an impact coefficient of 0.307, significant at the 1% level. 

The population aging index had a positive impact on the growth of pension insurance expenditure, with a coefficient of 2.550, significant at the 1% level. Population aging has increased the number of elderly people receiving pensions, greatly increasing the expenditure of China’s pension insurance fund. In recent years, some provinces even have a gap in the current period. Statistics [1] show that China’s urban pension funds spent 5130.14 billion yuan in 2020, while the income was 4437.57 billion yuan. 

Pension fund expenditure has a significant positive effect on economic growth, with an impact coefficient of 0.123, significant at the 1% level. The expenditure of pension insurance fund will increase the disposable income of the elderly and stimulate the consumption demand of the elderly, especially the demand for elderly care services, such as the elderly care demand, community medical care demand, and the demand for elderly supplies. Health and pension consumption of the elderly will show a growing trend in the future. 

In Model 3, the effects of population aging and endowment insurance expenditure were added to test the impact on economic growth. The impact coefficient of endowment insurance expenditure was positive and significant, while the impact of population aging index was no longer significant, indicating that it had a complete intermediary effect. 

The mediation effect test results show that pension expenditure has a complete intermediary effect on the relationship between population aging index and economic growth. This also provides an alternative explanation path for China’s population aging to promote economic growth.

In Model 2, the urbanization rate, the population education level, and the natural population growth rate all had a significant positive impact on the economy. From the current situation of China, the pension insurance contribution rate of general urban workers is higher than that of rural areas, and the corresponding pension treatment of the corresponding urban elderly people is also relatively high. Consequently, a higher urbanization rate increases the endowment insurance expenditure. A higher level of population education level increases the attention paid to endowment insurance, and the enthusiasm of paying endowment insurance premiums are relatively high, leading to greater pension security after retirement. A higher natural population growth rate leads to greater pension spending. 

In Model 3, the urbanization rate, the population education level, and the natural population growth rate have a positive impact on the economic growth, and they are significant at the 1% level. Urbanization clearly plays an important role in promoting economic development. A higher level of population education leads to a higher quality of social human capital, contributing to economic growth. A higher natural population growth rate leads to a more complementary total population and more abundant labor resources, a major factor of economic growth.

### 4.4. Mediation Effect Test of Medical and Health Care Expenditure

Table 7 shows the mediating role of health expenditure in the relationship between population aging and economic growth. The population aging had a positive effect on medical and health expenditure at the 1% level, with an effect coefficient of 1 (Model 2). China’s medical and health expenditure increased significantly as it became an aging population society. 

Statistics show that the average annual growth rate of China’s medical and health expenditure was about 18% from 2008 to 2019, and in 2008, the medical and health expenditure accounted for 1.12% of the fiscal expenditure and then gradually increased to 6.78%.

Population education and natural population growth promote health and health expenditure. In addition, the higher the level of population education, the more attention to health services is paid, and the higher the demand for national health resources investment. A higher natural population growth rate will inevitably lead to the expansion of the total scale and, naturally, a greater demand for medical and health expenditure. Therefore, health expenditure has a positive impact on economic growth, with an impact coefficient of 0.179, significant at the 1% level. Thus, an increase in health spending can boost economic growth. Statistics also show that, compared with 2008, the number of medical and health institutions in China increased by about 13%, health personnel increased by about 78%, and the total health cost increased by three times. On the one hand, the considerable health costs promote the growth of medical consumption and promote the development of medical and health services; on the other hand, medical employment and household income level also increase. In addition, the development of the medical service industry can effectively improve the health of residents, as it can supply more high-quality labor for economic growth. At the same time, two explanatory variables of population aging index and health care expenditure were added to Model 3, and the population aging index had no significant effect. 

### 4.5. Analysis of Regional Heterogeneity Tests for Five-Point Samples 

The level of population aging varies significantly in different regions of China, so the entire sample was divided into three regions for sample testing and to analyze the impact of population aging on economic growth in different regions.

The regression results of the eastern, central, and western population aging index on economic growth, pension expenditure, and health expenditure are arranged in Table 8.

Considering the findings (Table 8), a positive impact of population aging on economic growth exists in the eastern and central regions, and its influence coefficients are 0.310 and 0.560, indicating that population aging has the greatest effect on economic growth in Central China. However, the impact on the economic growth of Western China is not significant, and the impact coefficient is only 0.165. The population aging in Western China is mild among the three regions, and the proportion of children in Western China is high to a certain extent. The impact of the population aging index on economic growth is not obvious. 

Secondly, population aging has a significant positive impact on the endowment insurance expenditure, among which the western influence coefficient is the largest, with 2.931, followed by the east, where it is 2.251. In the central part, the influence coefficient is 1.635. It can be seen that, in the central and western regions, the positive impact of population aging on endowment insurance expenditure is significant. 

The positive impact of population aging on health care expenditure was significant in all three regions. The influence coefficient is the largest in the western region, followed by the central region and the eastern region. Furthermore, the population aging index has a greater effect on the social security expenditure in the economically underdeveloped areas. 

The results in Table 9 and Table 10 show that the pension insurance expenditure and healthcare expenditure both had a positive impact on economic growth. It should be noted that the central region had the largest impact coefficient, followed by the eastern region and the western region. 

Thus, the findings show that the intermediary effect of pension fund expenditure is significant (Table 8 and Table 9). Combining Table 8 and Table 10 shows that the mediation effect of Medicare funds in various regions was significant. Meanwhile, the Bootstrap sampling method was tested for each mediation effect, which showed a complete mediation effect. The findings validate the results of the full-sample analysis presented in the paper.

## 5. Discussion and Conclusions

This paper uses the data of 31 provinces (autonomous regions and municipalities directly under the Central Government) from 2008 to 2019 and uses the entropy method to comprehensively calculate the degree of population aging in 31 provinces in China in terms of the breadth, depth, and speed of population aging, and the regional differences are compared and analyzed. 

A regression model was applied to study the relationship between population aging, endowment insurance expenditure, medical and health expenditure, and economic growth. Empirical research results show the following:

(1) There are significant differences in the degree of population aging among regions. The eastern region has the highest degree of population aging, with the lowest degree in the west. The heavy burden of children can effectively alleviate the pressure of population aging in the future.
(2) The aging population has a significant positive effect. In addition, the endowment insurance expenditure and medical and health expenditure have a positive effect. However, there are more obvious differences in this effect between regions. The economic growth of the more developed areas is more significant compared with economically backward areas, which have high levels of endowment insurance expenditure and medical and health spending.
(3) The role of population aging on economic growth can play an intermediary role through endowment insurance expenditure and medical and health care expenditure. Different from some scholars’ view that population aging has a negative impact on the economy, the empirical results confirm that the positive impact of population aging on China’s economic growth is greater than its negative impact. Its internal mechanism is that population aging promotes economic growth by increasing endowment insurance and medical and health expenditure.

The comprehensive level of China’s population aging was calculated in multiple dimensions, showing the influence mechanism of population aging on China’s economic growth, which has enlightening policy implications for the establishment of a benign interaction between social security and economic development and the overall coordination of regional economic development. 

First, the Chinese government should improve birth policy in China and encourage the birth of two and three children, especially in the eastern region, which suffers severe population aging. It is also necessary to formulate strong relevant supporting policies to encourage people to have children and fundamentally curb the year-by-year decreasing trend of the labor force and population. At the same time, the Chinese government should accelerate the implementation of a delayed retirement system, gradually raise the legal retirement age, implement differentiated retirement pension policies in different regions, and ensure a sufficient amount and structural balance of labor resources. 

Second, it is necessary to improve the social security system, especially the endowment insurance and medical insurance systems. In recent years, the Chinese pension insurance fund has faced severe challenges in financial sustainability. In addition to increasing public financial investment, the state-owned capital transfer should enrich endowment insurance funds. Furthermore, the government should improve the national pension adjustment system, improve and stabilize residents’ future pension expectations, and improve the level of pension consumption of the elderly. At the same time, China should improve the medical service environment and actively promote the development of medical and health care undertakings with more preferential policies. This would allow for increased investment in medical and health care. 

Finally, in view of the differences in population aging by region, it is necessary to improve the construction of a multi-level labor market. Thus, the labor force could transfer freely between urban and rural areas, regions, and industries and balance the supply and demand of the labor force in the three regions. At the same time, China should focus on increasing old-age insurance and the medical and health care expenditure in the central and western regions. The sustainable economic growth in the central and western regions is promoted through the intermediary effect of old-age insurance and medical insurance expenditure.

Despite the valuable findings, this investigation has a few limitations. Firstly, in future investigations, it will be necessary to extend the list of variables that affect population aging and the country’s economic growth. Thus, it is necessary to analyze government efficiency, well-being, the public health system, globalization, and environmental pollution. In addition, the study does not consider the cointegration between the public health system and government efficiency, which allows for the allocation of appropriate incentives to improve the population’s well-being without decreasing economic development. Furthermore, it is necessary to increase the objects of the investigation, which would enable a comparison between countries/cities and the identification of best practices that improve the population’s well-being.

## Figures and Tables

**Table 1:** Index system and attributes of population aging.

Level 1 Indicators	Secondary Indicators	Attribute
The breadth of the population is aging	Population coefficient of the elderly	forward pointer
	The proportion of children’s population	negative indicators
	Old and young than	forward pointer
The population is aging deeply	Senior support ratio	forward pointer
	Child support ratio	forward pointer
The rate of an aging population	Growth rate in the elderly population	forward pointer

**Table 2:** Variables and definitions.

Type of Variable	Variable	Symbol	Meaning
explained	economic growth	VGDP	Regional GDP/regional total population
explanatory	Population aging index	AoP	The index of regional population aging calculated by the entropy method
metavariable	Pension insurance expenditure	PFE	Pension insurance fund expenditure
	Medical and health expenditure	MPE	Financial expenditure on medical care and health care
controlled	Urbanization rate	UBR	Town population/Total population
	Demographic education	PEL	College or above/6 and above
	Natural population growth rate	N PGR	(Number of births within year-number of deaths within year)/average year

**Table 3:** Population aging index of provinces and municipalities from 2008 to 2019.

Area	2008	2009	2010	2011	2012	2013	2014	2015	2016	2017	2018	2019
Beijing	0.466	0.450	0.430	0.411	0.387	0.384	0.381	0.505	0.537	0.550	0.477	0.497
Tianjin	0.561	0.479	0.455	0.425	0.459	0.514	0.516	0.456	0.494	0.480	0.477	0.541
Hebei	0.348	0.356	0.335	0.320	0.379	0.368	0.376	0.423	0.450	0.484	0.520	0.526
Liaoning	0.487	0.498	0.473	0.454	0.421	0.451	0.568	0.572	0.587	0.633	0.677	0.712
Shanghai	0.620	0.709	0.495	0.343	0.447	0.513	0.415	0.613	0.608	0.651	0.681	0.735
Jiangsu	0.491	0.497	0.463	0.440	0.483	0.517	0.495	0.535	0.569	0.581	0.596	0.625
Zhejiang	0.436	0.460	0.390	0.340	0.367	0.396	0.414	0.488	0.501	0.538	0.544	0.593
Fujian	0.397	0.401	0.337	0.288	0.361	0.320	0.294	0.389	0.422	0.373	0.381	0.402
Shandong	0.392	0.391	0.420	0.441	0.416	0.449	0.471	0.473	0.471	0.535	0.641	0.641
Guangdong	0.308	0.299	0.277	0.259	0.290	0.297	0.357	0.295	0.307	0.313	0.344	0.346
Hainan	0.364	0.349	0.298	0.259	0.300	0.346	0.300	0.349	0.341	0.328	0.330	0.388
Shanxi	0.327	0.326	0.316	0.310	0.323	0.322	0.358	0.376	0.356	0.366	0.440	0.446
Jilin	0.388	0.368	0.364	0.359	0.305	0.448	0.436	0.468	0.459	0.528	0.521	0.568
Heilongjiang	0.382	0.353	0.343	0.331	0.385	0.378	0.409	0.497	0.550	0.541	0.535	0.626
Anhui	0.432	0.395	0.409	0.416	0.407	0.423	0.416	0.458	0.470	0.541	0.534	0.564
Jiangxi	0.333	0.323	0.315	0.305	0.336	0.378	0.377	0.372	0.393	0.396	0.390	0.407
Henan	0.317	0.376	0.351	0.350	0.353	0.360	0.352	0.411	0.420	0.446	0.448	0.468
Hubei	0.413	0.412	0.410	0.409	0.447	0.388	0.419	0.468	0.480	0.500	0.509	0.528
Hunan	0.422	0.456	0.428	0.413	0.451	0.415	0.441	0.460	0.485	0.489	0.506	0.530
Nei Monggol	0.326	0.350	0.299	0.267	0.343	0.364	0.399	0.399	0.395	0.467	0.392	0.421
Guangxi	0.382	0.374	0.378	0.383	0.375	0.375	0.392	0.403	0.397	0.396	0.411	0.408
Chongqing	0.481	0.457	0.486	0.505	0.524	0.540	0.587	0.540	0.570	0.580	0.587	0.624
Sichuan	0.462	0.497	0.482	0.480	0.469	0.526	0.581	0.524	0.556	0.565	0.619	0.640
Guizhou	0.344	0.350	0.360	0.375	0.373	0.378	0.374	0.393	0.397	0.406	0.483	0.467
Yunnan	0.331	0.362	0.319	0.299	0.311	0.327	0.365	0.337	0.336	0.336	0.404	0.393
Xizang	0.275	0.291	0.221	0.180	0.247	0.215	0.253	0.241	0.215	0.274	0.240	0.271
Shaanxi	0.382	0.401	0.357	0.328	0.390	0.406	0.444	0.423	0.438	0.450	0.456	0.495
Gansu	0.336	0.336	0.345	0.357	0.374	0.349	0.360	0.387	0.409	0.417	0.467	0.457
Qinghai	0.288	0.286	0.252	0.230	0.312	0.292	0.286	0.292	0.296	0.332	0.299	0.355
Ningxia	0.276	0.287	0.237	0.209	0.304	0.298	0.269	0.314	0.328	0.352	0.372	0.385
Xinjiang	0.303	0.261	0.270	0.268	0.279	0.252	0.296	0.302	0.308	0.303	0.294	0.351

**Table 4:** Differences in the regional population aging index.

Level 1 Indicators		Population Aging Index	Breadth	Depth	Speed
East	average value	0.448	0.263	5.930	0.001
	standard error	0.107	0.087	1.081	0.005
	coefficient of variation	0.239	0.331	0.182	3.579
Central section	average value	0.418	0.218	6.701	0.001
	standard error	0.070	0.052	1.253	0.004
	coefficient of variation	0.169	0.241	0.187	3.017
West	average value	0.374	0.182	7.236	0.001
	standard error	0.097	0.053	0.994	0.004
	coefficient of variation	0.258	0.292	0.137	3.899

**Table 5:** Differences in population aging indicators in the various regions.

Secondary Indicators		Population Coefficient of Aging	The Proportion of the Children’s Population	Old and Young	Senior Support Ratio	Child Support Ratio	Old Year Growth Rate
East	average	0.105	0.141	0.819	14.032	18.874	0.021
	standard error	0.022	0.036	0.321	3.153	5.422	0.075
	coefficient of variation	0.208	0.254	0.393	0.225	0.287	3.579
Central section	average	0.101	0.166	0.644	13.936	22.993	0.018
	standard error	0.016	0.035	0.199	2.466	5.775	0.055
	coefficient of variation	0.156	0.210	0.308	0.177	0.251	3.017
West	average	0.091	0.191	0.505	12.791	26.787	0.015
	standard error	0.024	0.032	0.193	3.537	5.219	0.060
	coefficient of variation	0.262	0.168	0.382	0.277	0.195	3.899

**Table 6:** The intermediary role of the pension fund on the relationship between population aging and economic growth.

Variables	VGDP (Model 1)	PFE (Model 2)	VGDP (Model 3)
constant	3.066 ** (38.237)	18.924 ** (91.447)	0.735 * (1.980)
AoP	0.307 ** (4.216)	2.550 ** (13.563)	−0.007 (−0.081)
UBR	0.660 ** (5.841)	1.157 ** (3.965)	0.518 ** (4.725)
PEL	0.531 ** (10.503)	0.311 * (2.382)	0.492 ** (10.186)
N PGR	0.130 ** (5.430)	0.137 * (2.214)	0.113 ** (4.945)
PFE			0.123 ** (6.413)
sample capacity	372	372	372
adjust R^2^	0.742	0.632	0.767
F	F(4367) = 267.819, *p* = 0.000	F(4367) = 160.519, *p* = 0.000	F(5366) = 245.911, *p* = 0.000

Note: ** *p* < 0.01; * *p* < 0.05; Model 1 explains the total effect of population aging on economic growth; Model 2 explains the impact of population aging on pension fund expenditure; Model 3 explains the intermediary effect of pension fund expenditure; F: F statistic.

**Table 7:** Mediating role of health expenditure in the relationship between population aging and economic growth.

Variables	VGDP (Model 1)	MPE (Model 2)	VGDP (Model 3)
constant	3.066 ** (38.237)	7.607 ** (36.905)	1.704 ** (11.011)
AoP	0.307 ** (4.216)	1.991 ** (10.629)	−0.049 (−0.666)
UBR	0.660 ** (5.841)	−0.243 (−0.838)	0.704 ** (6.999)
PEL	0.531 ** (10.503)	0.415 ** (3.194)	0.456 ** (10.023)
N PGR	0.130 ** (5.430)	0.273 ** (4.426)	0.081 ** (3.719)
MPE			0.179 ** (9.922)
sample capacity	372	372	372
adjust R^2^	0.742	0.350	0.796
*F*	F(4367) = 267.819, *p* = 0.000	F(4367) = 50.879, *p* = 0.000	F(5366) = 290.839, *p* = 0.000

Note: ** *p* < 0.01; Model 1 explains the total effect of population aging on economic growth; Model 2 explains the impact of population aging on pension fund expenditure; Model 3 explains the intermediary effect of pension fund expenditure; F: F statistic.

**Table 8:** Results of sub-sample regression on the effects of population aging.

Sample Book	East	Central Section	West
Sample capacity	132	96	144
V GDP			
AoP	0.310 * (2.468)	0.560 ** (3.913)	0.165 (1.193)
*R^2^*	0.693	0.729	0.611
*F*	F(4127) = 75.008, *p* = 0.000	F(491) = 64.853, *p* = 0.000	F(4139) = 57.141, *p* = 0.000
PFE			
AoP	2.251 ** (6.217)	1.635 ** (5.656)	2.931 ** (9.991)
*R^2^*	0.338	0.686	0.773
*F*	F(4127) = 17.744, *p* = 0.000	F(491) = 52.889, *p* = 0.000	F(4139) = 123.023, *p* = 0.000
MPE			
AoP	1.090 ** (2.923)	1.456 ** (4.340)	2.622 ** (9.218)
*R^2^*	0.101	0.519	0.600
*F*	F(4127) = 4.683, *p* = 0.001	F(491) = 26.661, *p* = 0.000	F(4139) = 54.705, *p* = 0.000

Note: ** *p* < 0.01; * *p* < 0.05%; R^2^: R-squared; F: F statistic.

**Table 9:** Sub-sample regression results of the intermediary effect of pension funds.

Variable	East	Central Section	West
constant	−0.652 (−1.429)	−2.897 ** (−3.721)	1.159 (1.404)
A oP	−0.157 (−1.366)	0.025 (0.199)	−0.069 (−0.385)
PFE	0.208 ** (8.391)	0.327 ** (8.354)	0.080 * (2.019)
UBR	0.709 ** (3.070)	−0.294 (−1.370)	0.319 (1.701)
PEL	0.434 ** (5.054)	0.531 ** (5.569)	0.433 ** (4.907)
N PGR	0.096 ** (2.798)	0.064 ** (3.125)	0.020 (0.249)
sample capacity	132	96	144
adjust R^2^	0.802	0.846	0.619
*F*	F(5126) = 106.882, *p* = 0.000	F(590)=105.066, *p* = 0.000	F(5138) = 47.540, *p* = 0.000

Note: ** *p* < 0.01; * *p* < 0.05%; F: F statistic.

**Table 10:** Results of sub-sample regression of the mediation effect of health expenditure.

Variable	East	Central Section	West
constant	1.466 ** (9.161)	0.362 (1.608)	1.504 ** (3.904)
A oP	0.045 (0.495)	0.033 (0.393)	−0.211 (−1.252)
MPE	0.243 ** (11.743)	0.362 ** (15.227)	0.144 ** (3.623)
UBR	0.917 ** (4.567)	0.408 ** (2.667)	0.530 ** (2.790)
PEL	0.373 ** (5.005)	0.378 ** (5.485)	0.375 ** (4.416)
N PGR	0.062 * (2.056)	0.003 (0.192)	0.009 (0.124)
sample capacity	132	96	144
adjust R^2^	0.852	0.923	0.642
*F*	F(5126) = 152.272, *p* = 0.000	F(590) = 229.866, *p* = 0.000	F(5138) = 52.326, *p* = 0.000

Note: ** *p* < 0.01; * *p* < 0.05%; F: F statistic.

## Data Availability

The data presented in this study are openly available in [1].
